# Multi-phase functionalization of titanium for enhanced photon absorption in the vis-NIR region

**DOI:** 10.1038/srep15354

**Published:** 2015-10-19

**Authors:** Pooja Thakur, Bo Tan, Krishnan Venkatakrishnan

**Affiliations:** 1 Department of Aerospace Engineering, Ryerson University, 350 Victoria Street, Toronto M5B 2K3, Canada; 2 Department of Mechanical and Industrial Engineering, Ryerson University, 350 Victoria Street, Toronto M5B 2K3, Canada

## Abstract

Inadequate absorption of Near Infrared (NIR) photons by conventional silicon solar cells has been a major stumbling block towards the attainment of a high efficiency “full spectrum” solar cell. An effective enhancement in the absorption of such photons is desired as they account for a considerable portion of the tappable solar energy. In this work, we report a remarkable gain observed in the absorption of photons in the near infrared and visible region (400 nm–1000 nm) by a novel multi-phased oxide of titanium. Synthesised via a single step ultra-fast laser pulse interaction with pure titanium, characterisation studies have identified this oxide of titanium to be multi-phased and composed of Ti_3_O, (TiO._716_)_3.76_ and TiO_2_ (rutile). Computed to have an average band gap value of 2.39 eV, this ultrafast laser induced multi-phased titanium oxide has especially exhibited steady absorption capability in the NIR range of 750–1000 nm, which to the best of our knowledge, was never reported before. The unique NIR absorption properties of the laser functionalised titanium coupled with the simplicity and versatility of the ultrafast laser interaction process involved thereby provides tremendous potential towards the photon sensitization of titanium and thereafter for the inception of a “full spectrum” solar device.

The realisation of a “full spectrum” photovoltaic cell is still a distant dream and researchers continue to earnestly pursue it. Considering that visible and Near Infrared (NIR) radiation comprise of about 44% and 40% of the solar irradiation respectively on ground level when the sun is at its zenith, exploiting materials that are capable of harvesting sun light over a wavelength range of 400–1400 nm is more desirable for increase in the overall photovoltaic conversion^1^. Conventional solar cells efficiently absorb only the visible radiation, which comprises of about 44% of the solar spectrum^1^, but the narrow band gap of silicon renders most of the conventional solar cells to be ineffective absorbers of Near Infrared photons^2^. As such this part of the solar energy goes untapped and is wasted in the form of heat. To enhance the performance characteristics of a solar cell, it is essential to thus explore novel photovoltaic materials capable of tapping photons over the visible as well as the NIR range of the solar spectrum.

While there has been tremendous progress in finding photovoltaics materials that are good visible light absorbers, efficient harvesting of infrared photons has still not been feasible. The earliest attempt to study the collection of infrared radiation was done when the process of up-conversion was first suggested for the purpose of infrared detectors^3^. However, it was not until three decades later that this concept was further explored for potential photovoltaic application^4^, where typical up-convertors consisting of active ions set in a host material were integrated into solar devices. More recently, the unique optical properties of nanostructured materials offered new possibilities in the search for full spectrum solar harvesting materials. Incorporation of lanthanide nanocrystals, nanoshells and other rare-earth ions, as well as the fabrication of Single Walled Carbon Nanotubes (SWCNTs) and of late, polymer free SWCNTs, as potential near infrared absorbers^5^,^6^,^7^ have been done in an effort to efficiently broaden the optical gap to deeper NIR wavelengths. In recent times, the phenomenon of Localised Surface Plasmon Resonance (LSPR) has also been exploited to tune it to NIR radiation^8^. Gold nanoshells (spherical dielectric-metal core shell nanoparticles), embedded in a PbS Colloidal Quantum Dots film by a carefully choreographed method, have been used by *Paz-Soldan et al.* to fabricate a plasmonic exciton device^9^. This device is reported to have attained a peak 35% enhancement in absorption centered at 880 nm wavelength with the embedded nanoshells^9^ by judiciously balancing local optical enhancement, colloidal chemical stability and electronic insulation from surface recombination^9^. Research in the area of up conversion to NIR radiation and beyond is still being pursued and continuous effort is necessary to look for neoteric materials for enhanced trapping of visible and NIR photons.

Titanium on its own is a highly resistive and a reflective metal however, it is its naturally occurring oxide, titanium di-oxide that has generated a wide interest in the field of photovoltaics, post the discovery of its photo catalytic properties by Fujishima *et al.*^10^. It is extensively used as a mesoporous nanoparticle oxide layer in Dye Sensitized Solar Cells and strong UV light absorbing properties but is still plagued by a number of issues like the inefficient absorption of the visible and infrared part of the solar spectrum, lack of stability and reproducibility in its method of synthesis and inability to systematically tune and control the size and morphology of such nanostructures. There has still not been any substantial gain observed in the NIR region of the solar spectrum beyond 800 nm. As such, in an effort to improve the photon absorptive capacity of base titanium itself and transform its wavelength dependent absorptive response to a wavelength independent one, we have for the very first time, as per our knowledge, phase transformed and “functionalised” the optical properties of titanium by generating a novel multi-phased titanium oxide and thereby enhanced its absorption capability of visible and near infrared region photons. This multi-phased oxide of titanium, consisting of rutile and two other rare oxides of titanium, Ti_3_O and (TiO._716_)_3.76,_ was synthesised by a unique interaction process between ultra-short laser pulses and base titanium material, which facilitated its sensitization towards the photons of incident wavelength light. Calculated to have an average band gap of 2.39 eV, the multi-phase functionalized titanium oxide has shown a strong enhancement in absorbance in the vis-NIR region of the solar spectrum (400–1000 nm), with an absorption gain of about thirty times more than that of the base Ti in the wavelength range of 800–1000 nm. To the best of our knowledge, such a multi-phased oxide of titanium has been reported for the first time and such a degree of enhancement in absorption, especially in the NIR region of 800–1000 nm, has never been observed earlier for titanium and its oxides.

## Fabrication

The multiphase titanium oxide was generated by laser irradiation of pure bulk titanium. The titanium samples used for the experiments were cut from grade 2 pure titanium bars of 2 mm thickness. Ground finishing was followed by polishing to remove surface defects and contaminations. A diode pumped Yb-doped bulk femtosecond laser system, with a constant central wavelength of 1040 nm and pulse repetition rate ranging from 200 KHz to 26 MHz was used for the irradiation. The experiments were performed at a maximum average power of 16 W. A laser spot with diameter around 10 μm was scanned across the surface of the titanium sample using a two-axis galvoscanner.

Laser beam was scanned across the titanium surface to generate a number of parallel lines at a constant pulse width of 214 fs. The irradiation process was repeated with laser fluence set in the range of 0.8208 J/cm^2^ to 2.4614 J/cm^2^ and at laser scanning speeds of 50 mm/s, 100 mm/s and 200 mm/s. For a given pulse repetition rate and scanning speed, the effective number of laser pulses which interact with the surface area equivalent to that of the laser spot area, denoted as N_eff_, can be calculated as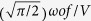, where *ω*_*0*_ is the laser spot radius, *f* is the repetition rate and *V* is the scanning speed.

With laser pulse duration of 214 fs, it was found that the absorbance of the irradiated titanium surface reached maximum at the scanning speed of 50 mm/s. Therefore, 50 mm/s was chosen as the optimum scanning speed. To test the effect of pulse width on the absorbance, the above mentioned experiments were repeated at pulse widths of 714 fs and 1428 fs while keeping the scanning speed constant at the observed optimum value of 50 mm/s.

## Material Characterization

Surface morphological study of the laser functionalised areas was conducted using Scanning Electron Microscopy (SEM), followed by the crystal lattice analysis using the High Resolution Transmission Electron Microscope (HRTEM). The phase composition was determined through X-ray Diffraction (XRD) analysis using a CuKα radiation source, conventional theta/2theta diffractometer. The average wavelength of the X-rays was 1.54184 Å and the profiles were obtained with a 2θ range of 30–78°. Further material characterization was done using Energy Dispersive X-ray Spectroscopy (EDX), X-ray Photoelectron Spectroscopy XPS and a micro-Raman using a 532 nm wavelength laser. The absorbance of the laser functionalised titanium region was measured for a broadband spectrum range of 400–1000 nm using a spectrophotometer.

## Results and Discussion

**Figure 1:**
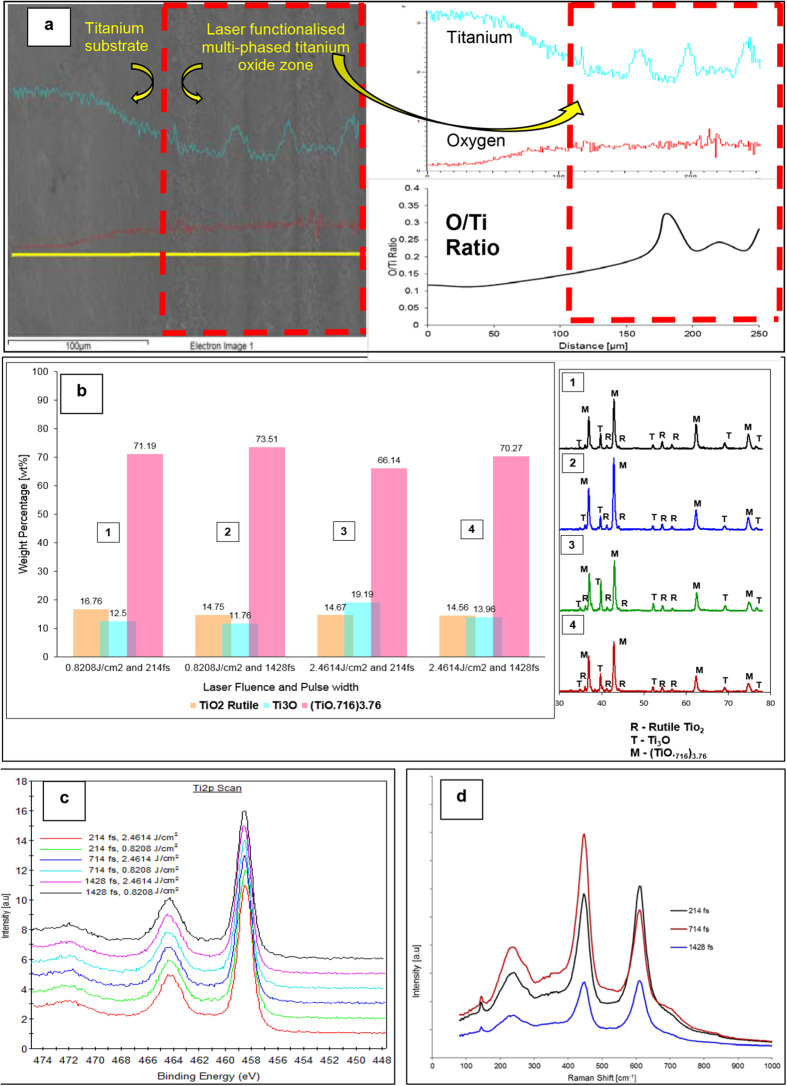
(**a**) EDX line scan showing the variation of oxygen and titanium content along the base titanium substrate and the laser functionalised zone. The O/Ti ratio plot indicates the obvious increase in areas where laser material interaction occurs (along the lines) (**b**) Wt. % of each of the three identified titanium oxides as obtained by the Rietveld fitting and the corresponding XRD patterns on the right, obtained at various laser parameters. (**c**) Ti2p XPS spectrum of the laser functionalised zone (**d**) Raman spectrum of the same showing peaks corresponding to rutile.

EDX analysis of the multi-phased titanium oxide of the laser functionalised titanium was conducted to quantitatively identify and characterise its principal material components. This study revealed the obvious increase in the oxygen content in the laser material functionalised zone Fig. 1(a) provides the EDX analysis of a laser functionalised titanium sample, where we see a very little oxygen content along the base Ti substrate area. However, an apparent increase in oxygen content (as observed by the greater value of the calculated O/Ti ratio in the accompanying plot) is seen along the area functionalised by the ultra-short laser pulses. Further careful study of the O/Ti ratio within the laser functionalised zone showed an increase in the oxygen content on the lines bought about by the oxidation at the site of the high temperature ultra-short laser material interaction process, while decreasing in intensity in between the functionalised lines.

Due to the inability of EDX to help us characterise and identify the individual constituent phases of the functionalised titanium oxide zone, XRD spectrum was obtained for the multi-phased oxide formed at various laser conditions. The resultant XRD pattern revealed that the oxide layer was composed of three distinct titanium oxides phases: (TiO._716_)_3.76_, TiO_2_ (rutile) and Ti_3_O. The basal titanium substrate showed peaks corresponding to α-titanium having highly discernible [002] and [101] orientations. To quantitatively characterise the multiple phases of the generated titanium oxide, Rietveld fitting of the XRD patterns was done.

Figure 1(b) shows the plot indicating weight percentage of each of the titanium oxides formed at a particular laser fluence and pulse width along with their corresponding XRD patterns obtained at each of those conditions. The most dominant phase (~70% weight percentage) was found to be a non-stoichiometric oxide of titanium having the molecular formula of (TiO._716_)_3.76_. This rare oxide of titanium, having an enlarged lattice when compared to that of the base Ti substrate, has a face centered cubic structure (fcc) and a lattice constant of a = 4.1966^11^. Another rare oxide of Titanium Ti_3_O, which has a hexagonal structure with lattice parameters of a = 5.15 and c = 9.56, accounts for about 15% of the weight^12^. The remaining is rutile TiO_2._

It was observed from the Reitveld fitting that at constant pulse width, with a decrease in laser fluence, the percentage composition of (TiO._716_)_3.76_ and rutile TiO_2_ increased while that of Ti_3_O decreased. Whereas, at constant laser fluence, as the pulse width decreased, the percentage composition (TiO._716_)_3.76_ decreased and the percentage composition of rutile TiO_2_ and Ti_3_O increased.

For an X-ray, with a Cu Kα radiation source, having a constant wavelength of 1.54060 Å, the calculated penetration depth is known to be approximately 7–8 μm at 2 θ values of 60–65°. The conspicuous absence of the Ti substrate peaks on the XRD plots of the samples shows that the X-rays were not able to penetrate the multi-phased titanium oxide layer. It can therefore be safe to ascertain the apparent thickness of the multi-phased oxide layer on the laser functionalised zones to be at least 7–8 μm. This absence of α-Ti peaks also signified that the femtosecond laser irradiation process did not lead to a partial but a “complete” oxidation of titanium.

To further corroborate XRD analysis, Raman and XPS spectrum was acquired. The Ti2p XPS spectrum showed the Ti 2p_3/2_ and Ti 2p_1/2_ binding energies to be approximately 458.5 eV and 464.5 eV respectively, as seen in Fig. 1(c). These peaks are characteristic of Ti^4+^ oxidation state and attributed to TiO_2_. Since XPS is a highly surface sensitive technique, it is possible that it could only characterize the top most thin oxide layer formed upon exposure to the ambient air (TiO_2_) and not the other lower oxides of titanium otherwise identified by XRD analysis. For the back scattering micro Raman spectroscopy analysis Argon gas ion laser having a central wavelength of 532 nm was used as excitation. The Raman plot of the non-functionalised titanium substrate area on samples was devoid of any peaks, which confirmed the absence of oxides on these areas. The measurements taken at ambient conditions of all the laser functionalised areas on the samples, showed three sharp peaks at the following given wave numbers: 240 cm^−1^, 443 cm^−1^ and 610 cm^−1^, as seen in the Raman spectrum given in Fig. 1(d). These peaks correspond to the characteristic peaks of rutile TiO_2_. As evidenced by the XRD results, no presence of anatase TiO_2_ was recorded by the Raman spectroscope in all the laser irradiated samples. The absence of the other two oxides of titanium (TiO._716_)_3.76_ and TiO_3_ on the Raman plots can be explained by the fact that neither of the two oxides are Raman active due to which they cannot be detected via the Micro Raman.

**Figure 2:**
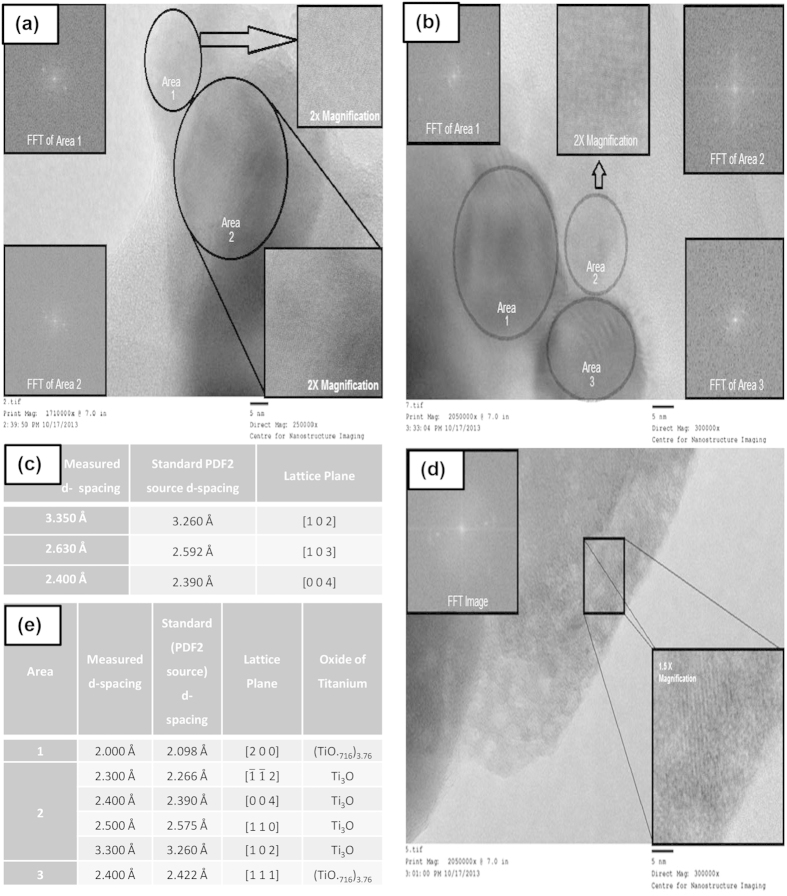
HRTEM image of (a) rutile TiO_2_ particle showing different planes in area 1 and 2, (b) hexagonal Ti_3_O, with the FFT and magnified image given in the inset, (c) d-spacing values of different planes of Ti_3_O as measured from figure (b) and standard (PDF2 source) values. (**d**) fcc-structured (TiO._716_)_3.76_ (area 1 and 3) and Ti_3_O (area 2), with the FFT image given in the inset (**e**) Measured and standard (PDF2 source) d-spacing values of fringes present in different marked areas of figure and the corresponding identified oxide of titanium.

HR-TEM was used to determine the, crystal lattice and verify the presence of the three titanium oxide phases identified in the XRD study. The results are given in Fig. 2. First, the d-spacing of the different lattice orientations were calculated using the Fast Fourier transform (FFT) image obtained by NIH’s ImageJ™ software. The measured d-spacing values were then compared to those listed in the standard Powder Diffraction File (PDF-2) database to see if the values agreed with any of the three identified titanium oxides.

In the Fig. 2(a), the d-spacing of the lattice fringes seen in area 1, calculated from the FFT image (inset) was found to be 2.40 Å. The two lattice planes seen in the FFT image of area 2 were calculated to have a d-spacing of 3.300 Å and 2.450 Å. These values seem to be consistent with the standard PDF2 values of [1 0 1] orientation plane (i.e. 2.487 Å) and [1 1 0] plane (i.e. 3.248 Å) of rutile phase of TiO_2_. Furthermore, the tetragonal shape of TiO_2_ is clearly visible in the marked area 2.

The lattice d-spacing of the FFT image of the fringes seen in Fig. 2(b) were calculated to be 3.350 Å, 2.630 Å and 2.400 Å. These d-spacing values are agreeable with the PDF2 values of hexagonal Ti_3_O oxide. The table in Fig. 2(c) shows the measured d-spacing values and the standard reference values for the corresponding lattice planes of Ti_3_O. [1 0 2], [1 0 3] and [0 0 4] lattice planes with the d-spacing values of 3.260 Å, 2.592 Å and 2.390 Å respectively. Figure 2(d) has three marked areas. The Fig. 2(e) shows the measured d-spacing values and the standard reference values from the PDF2 source and the titanium oxides to which they most correspond to.

**Figure 3:**
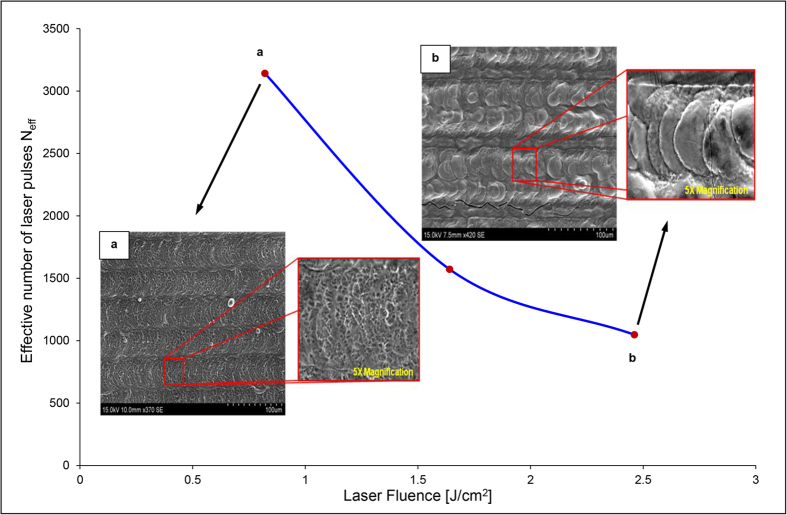
N_eff_ Vs Laser fluence plot with the corresponding SEM images of the laser functionalised zone at extreme condition of laser fluence.

In addition to material characterisation studies, surface morphology of the laser functionalised samples was examined via SEM to reveal the presence of a molten recast layer. This molten recast was more pronounced at higher laser fluence values and was seen to have ‘round platelet’ like formation. The overlapping of the platelets was seen to reduce with a decrease in the effective number of pulses due to fewer number of laser pulses hitting the same spot on the metal target before the laser moves on to the adjacent area. On the other hand, a ‘web-like’ surface was observed for areas functionalised at lower laser fluence. No removal of material was observed on any of the laser transformed samples. Figure (3) shows the variation of number of ultra-short laser pulses hitting the titanium sample surface as the fluence is changed. The corresponding change in surface morphology seen is indicated by the SEM images provided in the inset.

### Temperature analysis

In an attempt to understand the formation of the multiphase titanium oxide and simulate the thermal conditions caused by the ultrafast laser material interaction, first a theoretical study was conducted to calculate the final average surface temperature obtained at the centre of the laser-material interaction spot.

The following method was used to estimate the maximum temperature a target material attained when heated by a single laser pulse, at a given laser fluence. In this, the heat loss due to radiation and plume expansion was not considered for simplicity. It was also assumed that the energy of the laser would be absorbed much before the complete penetration of the heat wave happened. The modeling for this heat wave propagation was done according to the 1D heat conduction regime^13^,^14^:


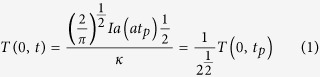


where T is the surface temperature obtained at the end of a laser pulse, at the center of the laser spot area. The rest of the parameters used were as follows: 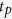 is pulse duration (pulse width), 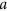 is the thermal diffusion coefficient, 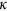 is the heat conduction coefficient and 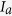 is the absorbed laser light intensity, which for a wavelength of 1040 nm was estimated to be^15^:


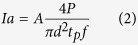


where, 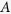 is the laser absorption coefficient, 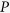 is the laser power, 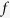 is the pulse frequency or repetition rate and 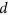 is the spot diameter. The laser absorption coefficient for titanium at 1040 nm was taken to be 0.448, thermal diffusivity as 0.090123 cm^2^/s and thermal conductivity as 0.219 (W/cmK).

Equation 1, applicable for a single laser pulse was then used to arrive at a second equation as described in the paper by *Gamaly et al.*, to calculate the average surface temperature 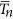 at the center of the laser spot after ‘n’ number of pulses^13^,^14^.


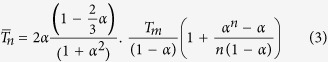


Where 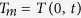 is the maximum temperature obtained at the end of the laser pulse at the laser spot center and 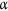 is a constant ratio given by 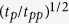, where t_*pp*_ = 1/f.

**Figure 4:**
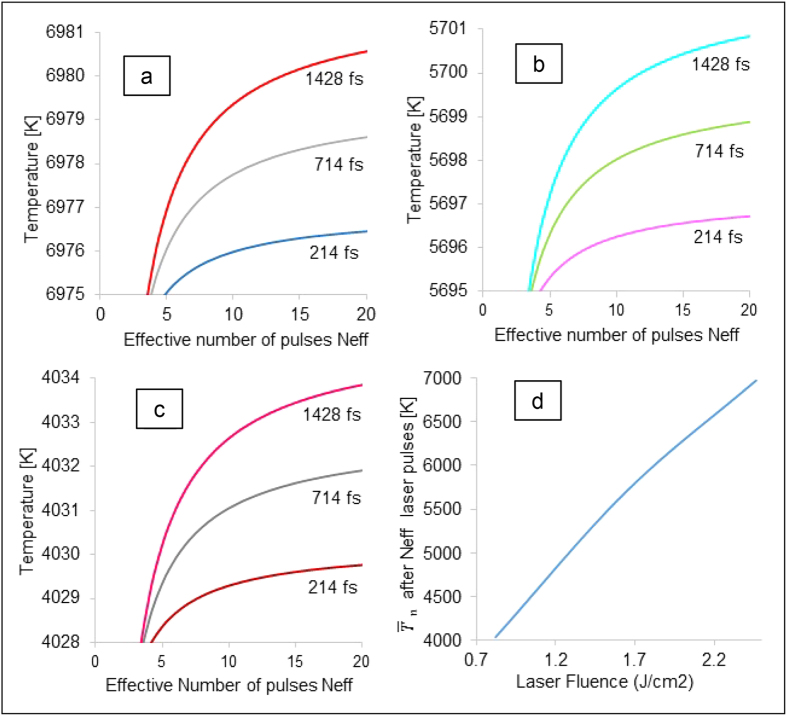
Average surface temperature calculated for the first twenty laser pulses at different laser fluence values: (a) 2.4614 J/cm^2^ (b) 1.6409 J/cm^2^ (c) 0.8208 J/cm^2^ and (d) Variation of average surface temperature with laser fluence.

This equation 3 was eventually used to calculate the theoretical average surface temperature at the laser spot center obtained for different laser parameters. A MATLAB™ code was written and executed to obtain a 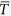
n Vs N_eff_ graph shown in Fig. (4). The value of 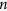 in equation 4 was substituted to be equal to that value of N_eff_ calculated for each specific laser parameters used, using the equation given in the earlier section.

Figure (4) shows the plots comparing the average surface temperature values (in Kelvin scale [k]) for different laser pulse widths at a given laser fluence at the end of N_eff_ number of laser pulses. A significant increase in average surface temperatures was seen with an increase in laser fluence (Fig. 4(d)). This increase in temperature was seen to occur very rapidly in the first few pulses, after which the values remained more or less constant, which implied that most of the energy transfer from the laser to the base metal substrate occurred during this time period. The plots also demonstrated that at constant laser fluence, increasing the pulse width results in a very minimal increase in temperature values (<10 K).

To further understand how this heat would dissipate away from the laser spot on the titanium surface, another temperature analysis was conducted using ANSYS™ APDL software. The problem was reduced to a very simple 3D transient heat conduction model by ignoring the negligible radiation and convection effects and considering a rectangular block of titanium with the following dimensions: 10mm × 10mm × 2mm and the following material properties: thermal conductivity 0.219 W/cmK, specific heat 0.523 J/g°C and density 4.15 g/cm^3^. For simplicity, the cumulative heating effect induced due to the scanning of the ultrafast laser pulse beam was ignored. As such, only a single laser-material interaction spot area was considered. A point heat source was introduced onto the surface of the titanium block for a time period analogous to the actual laser material interaction time for a unit laser spot area. This point heat source was assumed to generate a heat equivalent to the average surface temperature value 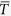 calculated above, at the end of N_eff_ number of laser pulses, at the center of the laser spot area. Subsequently the thermal heat diffusion along the surface and the depth of the titanium block was modelled to generate the temperature profile at various distances from the laser spot centre after several time periods. The analysis was performed for the three laser fluence conditions 2.4614 J/cm^2^, 1.6409 J/cm^2^ and 0.8208 J/cm^2^ at a constant pulse width of 214 fs. The transient temperature profile at other pulse widths was not modelled due to minimal temperature difference at the end of N_eff_ number of pulses, as seen in Fig. (4).

**Figure 5:**
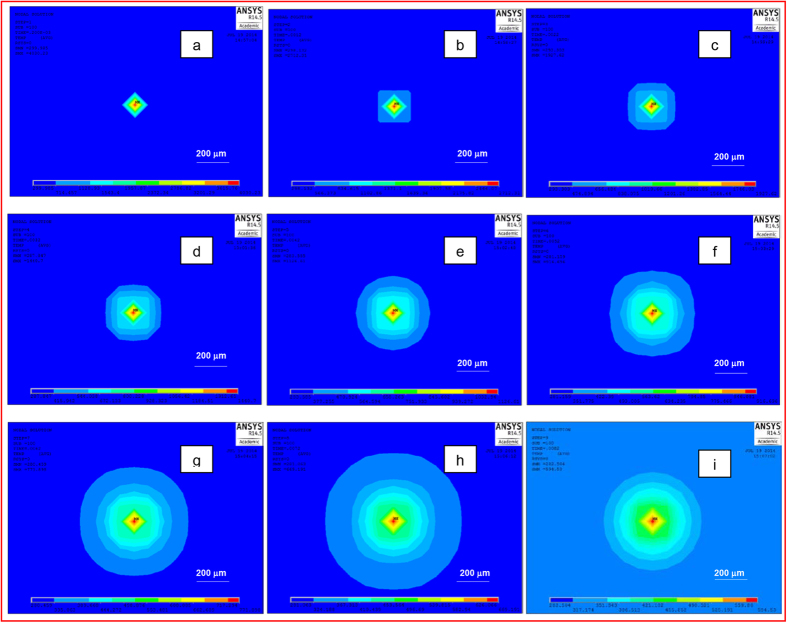
Thermal diffusion away from the laser material interaction spot for laser fluence of 0.8208 J/cm^2^ right after the laser beam is removed (a) 0.2 ms and a few time periods after the end of the interaction (b) 1 ms (c) 2 ms (d) 3 ms (e) 4 ms (f) 5 ms (g) 6 ms (h) 7 ms (i) 8 ms.

The contour plots in Fig. (5) display the progression of thermal diffusion away from a single laser-material interaction spot area with time, for a laser fluence of 0.8208 J/cm^2^. Similar contour plots were generated at other laser fluence values. To identify and explain the most probable oxide phase of titanium to have been formed at such thermal conditions, the Ti-O phase diagram was considered.

A careful observation of Ti-O phase diagram revealed the following about the three oxides identified earlier via XRD analysis on the ultrafast laser transformed multi-phased titanium oxide zone^11^:

i) Ti_3_O with a 25 at. % O is formed around about 673 K-773 K.

ii) (TiO._716_)_3.76_, having ~42 at. % O is a structural modification of the high temperature monoxide γTiO and is formed around 1793 K-2053 K.

iii) Rutile TiO_2_, stable at all temperatures, with a melting point of 2116 K is the richest O phase.

The oxidation of titanium at such elevated temperatures by the laser beam would evidently lead to the diffusion of oxygen atoms into the metal. The oxygen diffusion length would also be longer at higher fluence. Analysis of the temperature profile generated by the transient 3D model was done, followed by its correlation with the Ti-O phase diagram. The solidification path 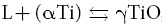, observed in an Ti-O phase diagram, could have been taken by the molten metal leading to the formation of (TiO._716_)_3.76_ through the diffusion of oxygen. The high oxygen content rutile would mostly form near the extreme surface by the following phase transformation: L ⇆ TiO_2_ and at a greater depths where diffusion of oxygen is limited, the second order phase transition (αTi) ⇆_2_⇆_3_O could have been the likely path leading to the formation of Ti_3_O.

**Figure 6:**
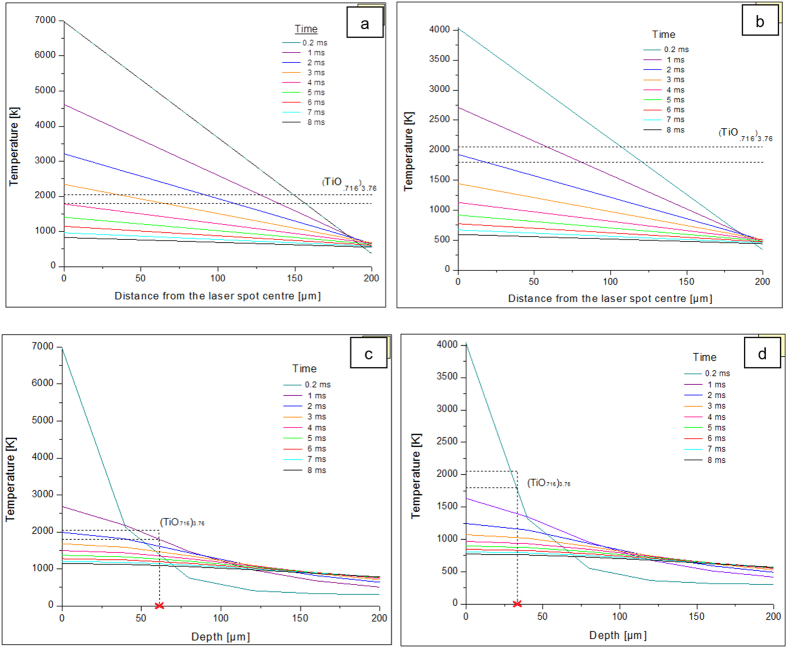
Temperature [K] Vs Distance from laser spot centre [μm] plot at laser fluence (a) 2.4614 J/cm^2^ (b) 0.8208 J/cm^2^. The possible (TiO._716_)_3.76_ formation temperature range is marked between the dotted lines in plot (**a**,**b**). Temperature [K] Vs Depth [μm] plot at laser fluence (**c**) 2.4614 J/cm^2^ (**d**) 0.8208 J/cm^2^. The possible (TiO._716_)_3.76_ formation temperature range is indicated between the dotted lines in plot (**c**,**d**), with the extrapolated x-intercept showing the maximum depth up to which it can form.

The temperature values generated by the transient model on the sample surface have been plotted in Fig. 6(a,b), up to a distance of about 200 μm away from the laser centre spot, at various time intervals. Similar plots showing the temperature values at different depths along the laser spot centre are given in Fig. 6(c,d).

A careful study of these plots showed that, the thermal conditions conducive for the formation of Ti_3_O at distances closer to the laser spot centre along the depth as well as on the surface, would develop only after about six to eight milliseconds post the end of the laser material interaction. It is however probable for Ti_3_O to have been formed much earlier, at greater depths, if adequate diffusion of oxygen could have occurred by then. However, due to the inability of our diffractometer to study the composition at such longer depths, it could not be verified by XRD analysis.

A plausible explanation for greater amount of Ti_3_O being formed at a higher fluence and shorter pulse widths (i.e. higher peak power), as determined by the Rietveld fitting, could be due to the presence of a much wider and deeper melt zone. The SEM image (b) given in the inset of Fig. (3) clearly indicates more melting at a higher fluence value of 2.4614 J/cm^2^, compared to lower fluence of 0.8208 J/cm^2^ seen in image (a) of Fig. (3). This accumulation of the melt zone having molten “platelets” as observed in the SEM images that could have caused a hindrance to the diffusion of oxygen is due to higher laser fluency of pulses hitting the sample during laser interaction. However, further study is required to explain this occurrence.

As mentioned earlier, (TiO._716_)_3.76_ is formed around 1793 K-2053 K. This temperature zone has been marked in Fig. (6) by dotted lines. On the surface, at regions closer than 100 μm from the laser spot centre, it was seen that such temperature conditions develop sooner, that is 1 ms after the cessation of the laser material interaction, at a lower fluence 0.8208 J/cm^2^. At higher fluence conditions of 2.4614 J/cm^2^, it would take 2–3 ms longer. This could explain the presence of greater amount of (TiO._716_)_3.76_ being generated on samples at 0.8208 J/cm^2^ laser fluence. Likewise, the marking of the temperature zone for the formation of (TiO._716_)_3.76_ along the depth, shown in Fig. 6(c,d) revealed that this non-stoichiometric oxide could form up to a depth of 60 μm at 2.4614 J/cm^2^ laser fluence condition and only up to 30 μm depth from the sample surface, at lower fluence of 0.8208 J/cm^2^. However, it is very unlikely that oxygen would diffuse to such depths, given the short (milliseconds) time scale.

### Absorption Properties

The optical properties of the irradiated Ti samples were studied by obtaining the absorbance spectrum for wavelengths in the range of 350 nm to 1000 nm, using a Spectroscope. The absorbance spectrum was plotted against the wavelength at constant laser fluence and then at constant N_eff_ values to compare the absorption properties of the multi-phased titanium oxide zone with that of the base untreated Ti substrate and subsequently calculate the effective increment in absorbance.

A number of interesting observations were made from the plotted graphs. A very pronounced increase in the absorbance with an increase in effective number of pulses was seen. The absorption intensity showed a steady increase as the wavelength increased from 350 nm to 1000 nm, reaching a maximum and plateauing after 750 nm. This increase was seen to be exponential until a wavelength of 600 nm in the electromagnetic spectrum. For wavelengths greater than 600 nm, a very distinctive increase in absorbance was observed, signifying that the laser irradiation process had a significant impact on the absorbance properties of the phase transformed titanium in the vis-NIR region of the electromagnetic spectrum. At a given laser pulse width, the direct proportionality of absorbance with the effective number of pulses and inverse variation with the laser fluence was observed.

**Figure 7:**
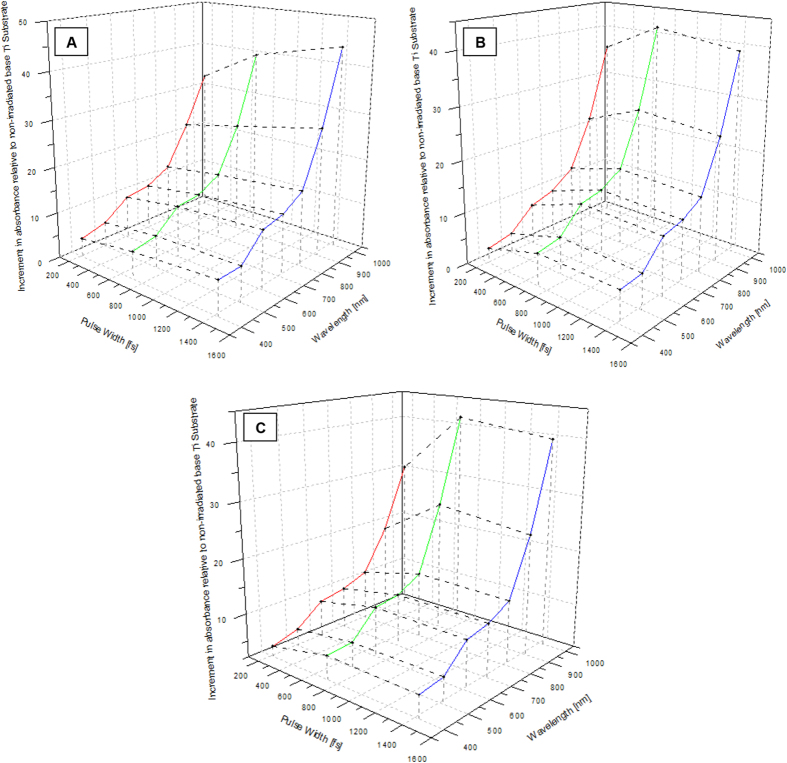
Increment in absorbance at different wavelengths for the three pulse width values of 214 fs, 714 fs, 1428 fs, at a constant laser fluence value of (a) 2.4614 J/cm^2^ (b) 1.6409 J/cm^2^ (c) 0.8082 J/cm^2^.

**Figure 8:**
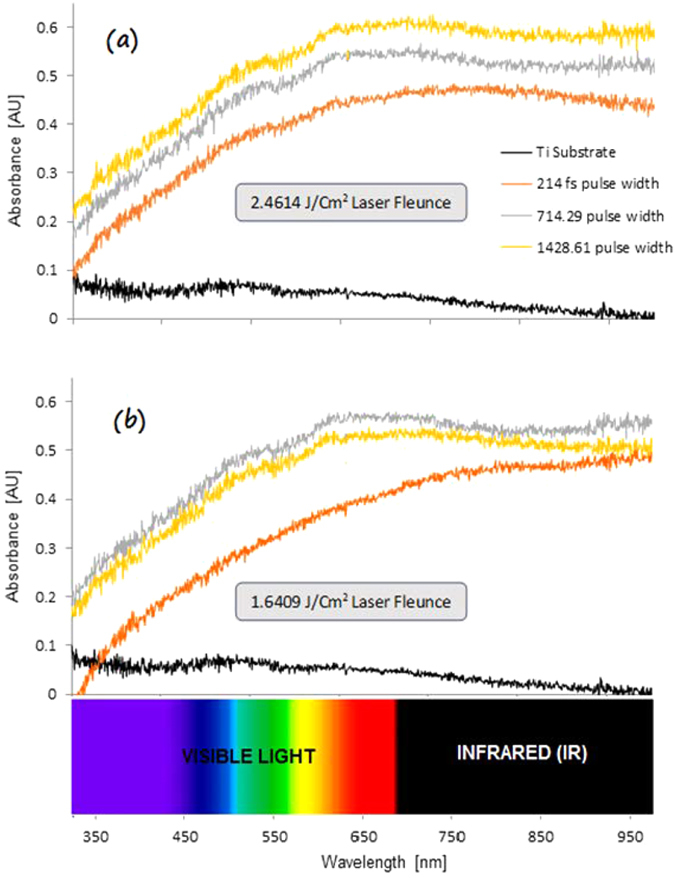
(**a**) Comparison of the absorbance spectrum of the laser transformed multi-phased titanium oxide zone at a constant laser fluence of 2.4614 J/cm^2^ and various pulse widths with that of the base titanium substrate. (**b**) Similar comparison at a laser fluence of 1.6409 J/cm^2^.

The spectral studies of the second set of experiments performed at various pulse widths revealed a similar increase in absorbance at greater wavelengths with respect to the base non laser transformed titanium surface. This can be seen in Figs 7. It was observed that the absorbance tended to generally increase as the laser pulse width became longer, as seen in Fig. 8(a). Further analysis of the plots revealed another important aspect. This increase in absorbance with pulse width showed a reverse trend from 714 fs to 1428 fs at a lower laser fluence values as seen in the graph 8(b). This might indicate the presence of a lower threshold value of laser fluence beyond which, the effect of increase in pulse width does not result in absorption enhancement but rather, veritably leads to its inhibition.

**Figure 9:**
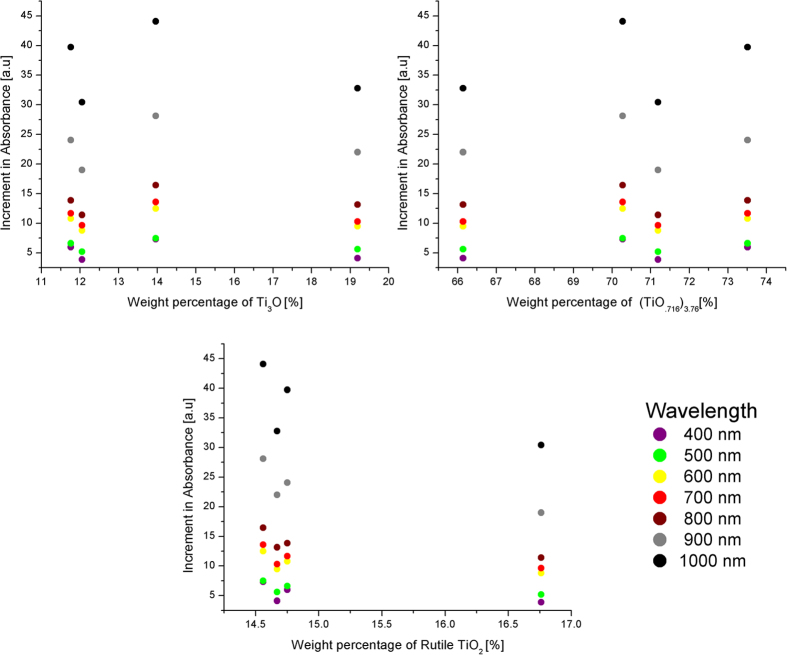
Increment in absorbance plotted against the weight percentage of the three oxides of titanium present in the multiphase laser transformed region: (a) Ti_3_O (b) (TiO._716_)_3.76_ (c) TiO_2._

Further comparative analysis was conducted by plotting the increment in absorbance obtained for each laser parameter against the Reitveld fitted weight percentage values of each of the titanium oxides calculated for the same conditions as given in Fig. 1(b). The individual plots for each oxide and the corresponding absorbance increment in the wavelength range of 400–1000 nm can be seen in Fig. (9). It was observed that the variation in the weight percentage of each of the three oxides had a much greater influence in the absorption at wavelengths longer than 800 nm. It was also observed that lower amounts of rutile and more of (TiO._716_)_3.76_ in the wavelength range 800–100 nm resulted in a considerable amount of gain in absorption for longer pulse durations. It is thus possible that the non-stoichiometric oxide phase of titanium (TiO._716_)_3.76_ plays a vital role in the enhancement of absorption in the NIR region.

### Band Gap Calculation

**Figure 10:**
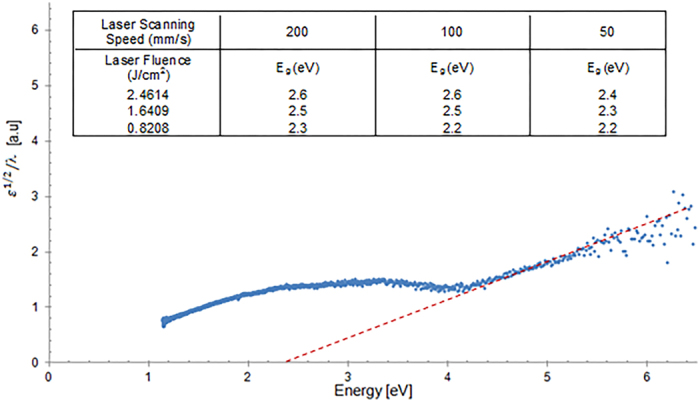
*ε*^1/2^/λ vs. E = *hc*/*λ* plot showing the measured band gap value to be 2.2 eV, at a laser fluence of 0.8208 J/cm^2^ and 50 mm/s scanning speed. The table given inset shows the band gap values generated at various laser parameters.

To further study the optical properties of the multi-phased oxide of titanium, its band gap, E_g,_ was calculated from the optical absorbance spectrum, 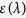. The Tauc’s equation was used to measure the band gap^16^:


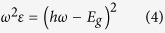


where, 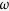 = 2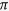/λ and 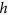 is the Plank’s constant. The intersection of a straight line extrapolated from the curve 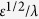 vs. 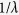 with the abscissa axis gives the value of the band gap. As shown in Fig. (10), the units of abscissa axis have been formatted to give the energy value E, in electron volts 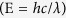, where 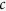 is the speed of light.

It was observed from the above calculation that the multi-phased titanium oxide exhibited an average band gap value of 2.39 eV, which is much lower than the bulk TiO_2_ band gap value of 3.2 eV. The most dominant oxide found in the multi-phased laser functionalised zone, (TiO._716_)_3.76_, renders its non-stoichiometric nature due to the presence of a number of crystallographic defects and vacancies. Any such disorders and imperfections in the structure are known to cause a narrowing of the band gap^17^,^18^. This could explain the lower band gap value of the laser functionalised zone.

## Conclusion

In this work we reported a novel oxide of titanium composed of multiple rare phases, which due to its notable vis-NIR absorption capabilities has the potential viability of being used as an intrinsic photon absorption component of next generation solar devices. The multi-phased titanium oxide, composed of three different oxide phases of titanium, was developed by a unique interaction between ultra-short laser pulses and the base Ti substrate. The composition of each of these phases was shown to be easily tuned in accordance with the ultrafast laser processing parameters, which induced photon absorption properties to the multi-phased titanium oxide material. The fabrication of this multi-phased oxide of titanium yielded to the transformative phase functionalization of the base Ti towards an average absorption gain of nearly ten times in the visible region to thirty times in the NIR region of the solar spectrum. Characterization studies were performed to identify the discrete titanium oxides, followed by a transient temperature analysis to understand the ultrafast laser processing conditions that were conducive for the oxide phase formation. The optical band gap of the generated rare multiphase oxide of titanium was averaged to be around 2.39 eV, which is about 25% lower than that rutile and 20% lower than anatase, the two most prevalent phases of TiO_2_ on which most of the next generation solar devices are based on. A window of opportunity exists to capitulate on the high photon absorptive properties of the generated multi-phased oxide of titanium and thereby transform and fine tune titanium into a highly photon sensitive material in the vis-NIR region. The significant absorption gain thus reported, especially in the NIR of the solar spectrum, by the unique phase functionalization of titanium can be availed in the continued pursuit over a “full spectrum” photovoltaic device.

## Additional Information

**How to cite this article**: Thakur, P. *et al.* Multi-phase functionalization of titanium for enhanced photon absorption in the vis-NIR region. *Sci. Rep.* **5**, 15354; doi: 10.1038/srep15354 (2015).
